# Monitoring technology for pest-plant interactions: The need for transdisciplinary research design

**DOI:** 10.1038/s44319-025-00489-3

**Published:** 2025-06-11

**Authors:** Emma Cavazzoni, Sabina Leonelli, Daniele Giannetti, Niccolò Patelli, Giacomo Vaccari, Lara Maistrello, Maria Cristina Pinotti

**Affiliations:** 1 Technical University of Munich, Munich, Germany; 2 University of Parma, Parma, Italy; 3 University of Modena and Reggio Emilia, Modena, Italy; 4 Provincial Phytosanitary Consortium of Modena, Modena, Italy; 5 University of Perugia, Perugia, Italy

**Keywords:** Evolution & Ecology, History & Philosophy of Science, Science Policy & Publishing

## Abstract

Transdisciplinary projects often fail to meet their goals due to institutional hurdles. Here are recommendations of how to achieve true transdisciplinarity in agricultural research.

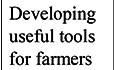

Automated monitoring and recognition are important tools for both pest management in agriculture and for the conservation of biodiversity. However, the use of such technologies for monitoring invertebrates has so far been limited owing to the difficulties of recording and identifying small animals. New and increasingly affordable technologies such as software and hardware with integrated AI (Van Klink et al, 2022; Mendoza et al, 2023), as well as drones or rovers, make it now possible to efficiently monitor invertebrates in the field (Sánchez Herrera et al, 2024; Sheard et al, 2024). These cutting-edge technologies hold great potential for identifying species, understanding population dynamics, and tracking the spread, abundance, density and phenology of specific pests in agricultural settings. However, their application in research and monitoring presents considerable challenges: the small size and behavior of insects, for example, require high-optical resolution that is not available with traditional camera-trap sensors. Even when such optical systems are integrated into monitoring instruments, other constraints persist, such as the precise visualization of key body parts necessary for accurate classification and taxonomic identification—for instance, the morphology of the aedeagus, the reproductive organ of male insects.

It is therefore essential to develop automated monitoring systems that account for the biology and behavior of diverse species, which requires expertise in computing, engineering, entomology and environmental science. For instance, addressing taxonomic challenges demands collaboration between engineers to develop high-optical-quality monitoring devices and entomologists to accurately classify insects. Similarly, translating monitoring data in agriculture into a defined action threshold that determines when a situation is severe enough to warrant intervention requires mobile monitoring technologies such as drones as well as an understanding of the behavior, developmental patterns and life cycle of insect species. Insight into the wider ecological relationships affecting pest-plant interactions, such as the role of microorganisms and environmental stressors, is similarly essential to ensure that the technology is able to measure the most significant variables.

## The need for transdisciplinary collaboration

Nevertheless, the biological aspect of technology development is often overlooked. Fueled by the ongoing interest in AI and computing, research projects tend to favor hardware development, often ignoring the application phase until later. Strictly enforced disciplinary boundaries further complicate effective integration, given the resilience of institutional requirements for individual researchers to publish within their own subfield, with little incentives and credit awarded for interdisciplinary collaborations.

Fueled by the ongoing interest in AI and computing, research projects tend to favor hardware development, often ignoring the application phase until later.

As a result, monitoring tools are frequently developed primarily by individuals without field experience and basic entomological knowledge, who—driven by the demands and pressures of their research environments—are compelled to prioritize technological aspects over practical functionality and entomological research. This disconnect creates a gap between technology development and field application, with researchers in computing and engineering disincentivized from assisting entomologists as well as farmers and small-to-medium agricultural businesses. Consequently, many technologies run the risk of becoming more of a technical showcase rather than a practical solution.

… many technologies run the risk of becoming more of a technical showcase rather than a practical solution.

In this comment, we argue for the crucial need of genuine transdisciplinary collaboration in agricultural research that leverages technologies and AI. Such collaboration requires the combination of diverse forms of academic expertise, as well as the integration of knowledge from stakeholders beyond academia. We begin by introducing the case study on which we base our reflections, the agricultural project Haly.Id. This was composed by scientists genuinely committed to transdisciplinary collaboration and actively striving to implement it, which precisely illustrates the kind of challenges they were confronted with in this mode of research. These obstacles can hinder the full achievement of the goals of monitoring projects, by shifting the focus toward the generation of large datasets and advanced technologies rather than finding solutions of real-world problems such as pest infestations. To mitigate this issue, we propose a set of guidelines for designing agriculturally-oriented research that successfully integrates monitoring technologies and AI. We conclude by highlighting how this approach must be supported by appropriate institutional recognition and funding structures to ensure its effectiveness.

## Challenges to transdisciplinary technology design

**Figure 1:**
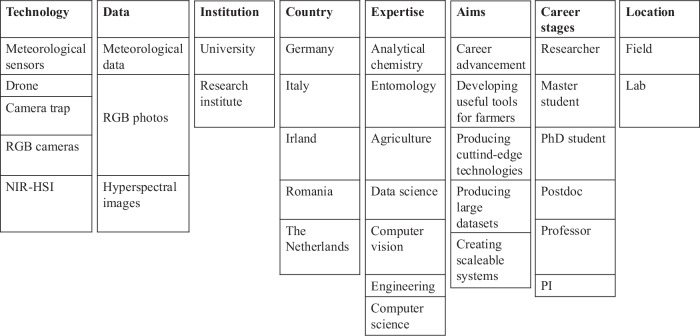
Haly.Id’s heterogenous and multidisciplinary structure.

Haly.Id (https://www.haly-id.eu) (Almstedt et al, 2024), a EU ERANET Information and Communication Technology project, took place between 2020 and 2024 in Northern Italy to monitor and manage the presence of the brown marmorated stink bug *Halyomorpha halys* in orchards. *Halyomorpha halys* is a highly invasive pest that feeds on fruits and has caused substantial damage and economic losses in southern Europe, eastern Asia and the USA (Leskey et al, 2012; Bariselli et al, 2016; Maistrello, 2024). Haly.Id showcased significant diversity in the technologies employed in the field and in the lab and in its social composition (Fig. 1). Working on a pear orchard, researchers adopted a drone for taking high-resolution videos and/or pictures of pear trees, which were subsequently analyzed by machine learning (ML) techniques to detect *H. halys* (Dinca et al, 2023; Betti Sorbelli et al, 2024; Giannetti et al, 2024). Other technologies included meteorological sensors (Betti Sorbelli et al, 2024), RGB cameras, a camera trap with a pheromone lure to attract and capture insects (Kargar et al, 2024), and a near-infrared hyperspectral imaging camera for identifying hidden pear damage (Ferrari et al, 2023).

Participants varied widely in terms of institutional affiliations and interests – some were from universities focused on research and academic publications, while others belonged to institutes and commercial companies centered on product development and commercialization. Geographically, they were spread across five European countries, and their expertise came from entomology, data science, analytical chemistry and engineering.

The Haly.Id project made substantive efforts to integrate its technological elements with entomological knowledge. For instance, it assessed the impact of drones on insects’ behavior, an aspect often overlooked in research using the same technology. Researchers conducted multiple studies to evaluate the suitability of drones for monitoring the presence of *H. halys* in orchards, concluding that drones, unlike human operators, do not cause the bugs to hide; instead, the wind they generate induces them to cling to surfaces. In addition, the drone monitoring focused on specific areas of the orchard, intentionally excluding empty spaces or trees that were dead or without leaves, where bugs are less likely to be found. Instead, the attention was placed on areas where insects could potentially cause damage, keeping their behavior in mind.

In a rare display of genuine transdisciplinary cooperation, Haly.Id involved not only academic entomologists but also experts beyond academia, such as farmers and regional plant protection services, incorporating their local knowledge into research. For instance, the research strand dealing with pears grown in the field and later analyzed in the laboratory depended on farmers for harvesting the fruits, technicians and plant protection operators. The latter also provided essential materials for Haly.id experiments, such as captured individuals.

In a rare display of genuine transdisciplinary cooperation, Haly.Id involved not only academic entomologists but also experts beyond academia…

Despite this ideal set-up, fully integrating local knowledge and entomological expertise proved challenging due to the central role of data and technologies, and the difficulty of reconciling varied approaches, disciplinary needs and individual goals while operating under the pressure of publishing (François et al, 2020). Like most projects of this kind, Haly.id was constrained by strict timelines and deliverables, and the dominant tendency to prioritize novel technological developments over complex social interventions (Leonelli, 2016; Falkenberg et al, 2023). At the same time, researchers had to allocate space for additional academic commitments as required by their institutions. This had implications that may run counter to the overarching interdisciplinary goals of transnational projects: in Italy, for instance, discipline-specific publications remain crucial for securing funding and academic career advancement (Pretolesi et al, 2025), particularly for early-career researchers whose evaluation and further recruitment depend on centralized state criteria.

Like most projects of this kind, Haly.id was constrained by strict timelines and deliverables, and the dominant tendency to prioritize novel technological developments over complex social interventions.

Thus, despite Haly.Id’s admirable efforts to integrate engineering, computational, entomological and non-academic knowledge from the outset of the project, the institutional environment of academic researchers involved in the project prioritized discipline-focused outcomes over integrated, cross-disciplinary results. Participants working on the technological aspects had to prioritize data collection and computational advances over finding ways to apply such methods towards monitoring and mitigating *H. halys* damage. The creation of extensive datasets and the development of cutting-edge technologies such as NIR-HSI and the drone system were considered benchmarks of success, often overshadowing the project’s practical goals (Cavazzoni and Leonelli, forthcoming).

A striking example of this tendency was the installation of cameras in the orchard to capture images every 10 min, tracking insect activity throughout the day. This approach generated far more data than the drone, which operated for just 30 min daily, or the camera trap, which took only two photos per day. While such high-frequency data collection offers potential for future applications, its necessity was questionable from an entomological perspective: *H. halys* often remains motionless for hours, making many of the captured images redundant. Moreover, the system was implemented less than a year before the project’s conclusion, leaving insufficient time to analyze or integrate the data into Haly.Id.

## Lessons from Haly.Id and similar projects

The project was highly effective in producing high-throughput tools for data collection which could encourage new expertise and creative engagement, yet there was little time to integrate the technologies and data produced with field and entomological expertise. Achieving such integration in ways that would support farmers in managing *H. halys* would require another round of funding through a follow-up project.

This case fits the Evolutionary Mismatch Hypothesis proposed by Li et al (2017), which highlights the disparity between the rapid advancement of technology and the slower pace of human adaptation. The pursuit of cutting-edge innovations—prioritized for their novelty rather than their functionality—exacerbates this mismatch and risks leading to knowledge narrowly shaped by the capabilities of specific tools and dependence on technology providers who have less incentive to ensure the quality, reliability or long-term scientific value of the data produced. The limited integration of local knowledge and zoological, ecological and ethological expertise then threatens to compromise research goals which, although based on technologies, are ultimately focused on the study of nature.

The challenges faced by Haly.Id reflect broader issues common to similar research efforts, where opportunities for effective collaborations across disciplines often remain confined to the early exploratory phases or emerge only as an add-on once technological components have been developed. As Burch et al (2023) point out, agricultural projects frequently struggle to incorporate social scientists as well as non-academic experts throughout all stages of research due to disciplinary divides, institutional barriers and funding mechanisms that neither encourage meaningful interdisciplinary collaboration nor reward it as career-enhancing. As a result, scientific and technological innovations often take priority, failing to account for real-world needs and leading to unforeseen social and environmental challenges with no attempt to adequately prepare for potential adverse outcomes. Similarly, Pooley et al (2014), writing on conservation science, highlight systemic obstacles to integrating ecological and social insights, including publication pressure, the demand for positive outcomes, tight timelines, the undervaluing of interdisciplinary outputs and rigid disciplinary boundaries. Because of broader environmental and social constraints, the development of hardware, software or modeling tools designed to capture and analyze entomological data may end up not systematically involving entomologists and non-academic experts, highlighting significant barriers to true transdisciplinary integration.

## Recommendations

To mitigate this situation and develop data-intensive technologies that incorporate entomological knowledge and can effectively tackle real-world problems, we outline key aspects that should be considered when designing agricultural research projects involving monitoring technology and AI. This would require cultivating an empirically grounded understanding of the biology, ecology and behavior of the target species; selecting technologies suited to environmental conditions, assessing both their functionality and impact; evaluating the biological and social effects of technological implementation before and after deployment; ensuring the usability and accessibility of technologies to farmers and field operators; evaluating the economic sustainability of the technological tools, for instance by analyzing the cost-benefit ratio from both a short and a long-term perspective, considering direct and indirect costs, and assessing whether farms of different sizes can afford such tools; and comparing the effectiveness of new monitoring technologies with conventional methods by measuring key performance indicators.

Effectively implementing these recommendations requires a sustained transdisciplinary approach, grounded in three key dimensions. First, interdisciplinarity that fosters meaningful integration of biological expertise with technologically-oriented disciplines such as data science or engineering. Second, transdisciplinary collaboration beyond academia, incorporating the local knowledge of stakeholders that possess firsthand experience and expertise in the field, such as farmers, technicians and plant protection services. Third, collaboration with social scientists and humanists specialized in cross-disciplinary and public engagement with science and technology, whose expertise can strongly support transdisciplinary work.

Indeed, technological experts, on their own, cannot effectively determine the most suitable technology without a clear understanding of the biological traits of the target species, the environmental and local conditions where it will be used, and how these tools interact with natural systems. Similarly, biologists and local experts require technological knowledge to assess which tools are most effective in gathering accurate data, and their specific technical features to make informed decisions when evaluating costs for practical implementation. Engaging diverse expertise throughout the development and construction phases of technological systems, addressing each aspect outlined in the guidelines, is essential for creating situated solutions to agricultural concerns and fostering long-term research efforts.

Engaging diverse expertise throughout the development and construction phases of technological systems […] is essential for creating situated solutions to agricultural concerns and fostering long-term research efforts.

This approach should be supported through institutional incentives, and yet the requirements of transdisciplinarity are often underestimated in the design and funding of large projects and fall outside the disciplinary organization of research assessment and credit structures. We emphasize the urgent need for improved funding, institutional incentives—or at the very least the removal of obstacles such as requirements for disciplinary publishing—and methodologies for transdisciplinary research that combines integration of biological knowledge and technological aspects, with engagement beyond academia.

Currently, research tends to focus on what is technically achievable rather than what is most useful given strategies already on the ground. This emphasis must change if monitoring technologies and AI are to effectively support solutions to agricultural challenges and food security. We suggest a shift in agricultural research towards prioritizing the development of technologies that build upon and enhance interventions that are already available. Technological advancements should be aligned with and strengthen existing forms of solutions in the field – an approach made possible through the integration of biological and technical expertise, as well as knowledge from non-academic stakeholders.

## Supplementary information

Peer Review File
