# Supplementary material for: Monitoring technology for pest-plant interactions: The need for transdisciplinary research design
Source: EMBO Rep. 2025 Jun 11;26(13):3236–40. doi: 10.1038/s44319-025-00489-3 (PMC12238487; doi:10.1038/s44319-025-00489-3)
Supplement: Supplementary file 1 — Peer Review File [file 44319_2025_489_MOESM1_ESM.pdf]

EMBO reports

# Monitoring technology for pest-plant interactions: The need for transdisciplinary research design

Emma Cavazzoni, Sabina Leonelli, Daniele Giannetti, Niccolò Patelli, Giacomo Vaccari, Lara Maistrello, and Maria Cristina Pinotti

*Corresponding author(s): Emma Cavazzoni (emma.cavazzoni@tum.de), Sabina Leonelli (sabina.leonelli@tum.de)*

**Review Timeline:**

| | |
|---|---|
| Submission Date: | 7th Apr 25 |
| Editorial Decision: | 9th Apr 25 |
| Revision Received: | 22nd Apr 25 |
| Accepted: | 19th May 25 |

*Editor: Holger Breithaupt*

**Transaction Report:** This article was editorially reviewed.
